# Impact of night shift work on sleep quality and mental health in hospital staff

**DOI:** 10.1192/j.eurpsy.2026.12099

**Published:** 2026-07-31

**Authors:** R. Hayat, S. Boughdadi, S. Karroumi, I. Adali, F. Manoudi

**Affiliations:** Ibn Nafis Hospital, Psychiatry, Mohammed VI University Hospital, Marrakech, Morocco

## Abstract

**Introduction:**

Night shift work, although necessary in the hospital sector, disrupts circadian rhythms, impairs sleep quality, and increases the risk of anxiety and depressive disorders. While this issue is well documented internationally, it remains understudied in Moroccan healthcare professionals. Addressing this gap is crucial, as understanding the mental health consequences of night work is key to improving staff well-being and patient care.

**Objectives:**

This study aimed to assess the impact of night shift work on sleep quality and mental health among healthcare staff at Mohammed VI University Hospital in Marrakech, by comparing night and day workers.

**Methods:**

We conducted a cross-sectional descriptive study among medical and paramedical staff at Mohammed VI University Hospital. All hospital staff were included in the study. Exclusion criteria included psychiatric disorders diagnosed prior to employment and pre-existing sleep disorders (e.g., sleep apnea, narcolepsy, chronic insomnia). An online questionnaire was distributed vi the hospital Whatsapp group; to collect sociodemographic and professional data, alongside the Pittsburgh Sleep Quality Index (PSQI) and the Hospital Anxiety and Depression Scale (HADS). Responses were collected over one month (22 February–22 March 2025). Participants were divided into two groups: night workers (> 4 night shifts/month) and day workers (≤4 night shifts/month).

**Results:**

Out of 279 questionnaires collected, 7 were excluded due to pre-existing sleep or psychiatric disorders. The final sample included 272 participants, of whom 76% (n = 212) were physicians and 24% (n = 60) nurses. Two groups were defined: 50.3% (n = 137) worked > 4 nights/month, and 49.6% (n = 135) worked ≤ 4 nights/month. Night shift workers had significantly poorer sleep quality compared to day workers, with higher mean PSQI scores (p = 0.005). Depression was more pronounced in this group, with higher mean scores (8.7 vs. 6.94) and a significantly higher prevalence of clinical depression (16.7% vs. 5.6%; p = 0.032). Correlation analysis revealed a significant association between poor sleep quality and increased levels of depression (r = 0.401, p < 0.001) and anxiety (r = 0.392, p < 0.001). These findings suggest that sleep disturbances related to night work may contribute to the worsening of anxiety-depressive symptoms among hospital staff.

**Conclusions:**

Night shift work negatively affects sleep quality and increases the risk of anxiety and depressive disorders among healthcare professionals. Improved scheduling, sleep hygiene strategies, and psychological support are needed to preserve staff well-being. These findings highlight the urgency of preventive strategies and institutional policies that prioritize the mental health of healthcare workers.

**Disclosure of Interest:**

None Declared

